# Novel beagle model of gastric local fibrotic target lesions for the evaluation and training of endoscopic techniques

**DOI:** 10.1186/s12876-023-03055-0

**Published:** 2023-11-27

**Authors:** Xiao-Jian He, Xiao-Ling Wang, Chuan-Shen Jiang, Dong-Gui Hong, Hai-Lan Lin, Yun-Ping Zheng, Han Li, Xin-Jiang Chen, Jian-Xiao Huang, Ling-Shuang Dai, Mei-Yan Liu, Bao-Xiang Luo, Dong-Liang Li, Da-Zhou Li, Wen Wang

**Affiliations:** 1 Fuzong Clinical Medical College, Fujian Medical University, Fuzhou, China; 2 Department of Digestive Diseases, 900TH Hospital of Joint Logistics Support Force, 156 North Road of West No.2 Ring, Fuzhou, 350025 China; 3 Department of Hepatobiliary Disease, 900TH Hospital of Joint Logistics Support Force, 156 North Road of West No.2 Ring, Fuzhou, 350025 China

**Keywords:** Animal model, Lesions with submucosal fibrosis, Endoscopic submucosal dissection, Preclinical evaluation and training, Advanced endoscopic techniques

## Abstract

**Background:**

Novel endoscopic techniques used in the treatment of gastric lesions with local submucosal fibrosis need preclinical evaluation and training due to safety limitations. Therefore, the purpose of our study was to establish an animal model of gastric local fibrotic target lesions and assess its feasibility in the evaluation and training of endoscopic techniques.

**Methods:**

In six experimental beagles, a 50% glucose solution was injected into three submucosal areas of the fundus, body, and antrum of the stomach to create gastric local fibrotic target lesions (experimental group). On post-injection day (PID) 7, the injection sites were assessed endoscopically to confirm the presence of submucosal fibrosis formation, and the dental floss clip traction assisted endoscopic submucosal dissection (DFC-ESD) procedure was performed on the gastric local fibrotic target lesions to confirm its feasibility after endoscopic observation. The normal gastric mucosa of six control beagles underwent the same procedure (control group). All the resected specimens were evaluated by histological examination.

**Results:**

All 12 beagles survived without postoperative adverse events. On PID 7, 16 ulcer changes were observed at the injection sites (16/18) under the endoscope, and endoscopic ultrasonography confirmed the local submucosal fibrosis formation in all ulcer lesions. The subsequent DFC-ESD was successfully performed on the 32 gastric target lesions, and the mean submucosal dissection time in the ulcer lesions was greater than that in the normal gastric mucosa (15.3 ± 5.6 vs. 6.8 ± 0.8 min; *P* < 0.001). There was no difference in rates of en bloc resection, severe hemorrhage, or perforation between the two groups. Histological analysis of the ulcer lesions showed the absence of epithelial or muscularis mucosae and extensive submucosal fibrous tissue proliferations compared with normal gastric mucosa. Overall, endoscopists had high satisfaction with the realism and feasibility of the animal model.

**Conclusion:**

We developed a novel animal model of gastric local fibrotic target lesions to simulate difficult clinical situations, which strongly appeared to be suitable for the preclinical evaluation and learning of advanced endoscopic techniques.

**Supplementary Information:**

The online version contains supplementary material available at 10.1186/s12876-023-03055-0.

## Introduction

Endoscopic submucosal dissection (ESD) has been applied in the excision of early mucosal cancer and submucosal benign tumors of the digestive tract, with the advantages of less trauma, less bleeding, and quick recovery [1, 2]. However, there are some difficult cases during the ESD operation, one of which is submucosal fibrosis. Submucosal fibrotic lesions have become an emerging clinical problem, which are considered to be closely related to ulcers, submucosal infiltration, and previous endoscopic procedures (e.g., previous biopsy sampling, trap sampling, endoscopic mucosal resection, etc.) [3–5]. And such lesions can be a technical challenge for the operator, as minor mismanagement can lead to serious surgical adverse events (e.g., delayed perforation). It is preferable that a more experienced physician perform ESD for cases with local submucosal fibrosis [6].

Many new methods have also been proposed to aid in the safety of ESD [7–10], such as dental floss clip traction, magnetic traction, suture pulley traction, or robotic arm-assisted endoscope. Although dental floss clip traction-assisted ESD has been widely performed in clinical practice for its feasibility and cost-effectiveness [8, 11], there are few reports on the safety and efficiency of dental floss clip traction systems or other traction systems in the dissection of gastric local fibrotic lesions. In addition, there is a demand to introduce novel or modified endoscopic techniques to assist ESD in the lesions with local submucosal fibrosis with a shorter operative time and a lower adverse event rate. Before these techniques are routinely used in clinical practice, adequate evaluations on their safety and efficiency issues are required in the preclinical study.

Given the possibility of higher procedure-associated discomfort and adverse event risks brought by the new techniques of ESD in local fibrotic lesions, the special preclinical training programs are necessary but not currently suitable to be carried out on human patients. The endoscopic simulators have been regarded as a bridge between theoretical teaching and human training by Eastern and Western endoscopic experts [12, 13]. Several studies have also confirmed that endoscopic simulators, including box simulators, ex vivo or in vivo animal models, and virtual reality simulators, could accelerate the learning curve for various endoscopic procedures [14–17]. Animal models are the most commonly used type of simulator for their anatomical structures and physiological feedback (e.g., bleeding, inflammation, secretion, tactile sensation, etc.), similar to humans. Besides, live animal model training could facilitate the acquisition of skills in complex endoscopic techniques and promote the application of new techniques in clinical practice [18, 19]. Although there were lots of experimental models to simulate target lesions in the animal stomach [17, 20], only pseudopolyps or depressed mucosal defects without local submucosal fibrosis were described in the previous studies. There are no appropriate animal models used for preclinical evaluation and training of advanced endoscopic techniques in gastric local fibrotic lesions, which may impede the development of endoscopic techniques. Therefore, the aim of this study was to develop and assess a new live animal model of gastric local fibrotic lesions that could be used for the preclinical evaluation and training of new endoscopic techniques.

## Materials and methods

### Preparation of animal

Twelve healthy beagles (weights of 12–16 kg), divided into experimental (N = 6) and control animals (N = 6), were fasted for 48 h before endoscopic procedures were performed under general anesthesia. Initially, xylazine hydrochloride (0.01–0.02 mL/kg) was intramuscularly injected, and then an intravenous catheter was placed in the forearm vein for maintenance anesthesia. Subsequently, an endoscopic bite block was placed in the mouth, and the endoscopic procedure was performed. The Institutional Review Board approval was obtained from the 900TH Hospital of Joint Logistics Support Force (approval No. 2021-008).

### Creating gastric local fibrotic lesions

**Fig. 1:**
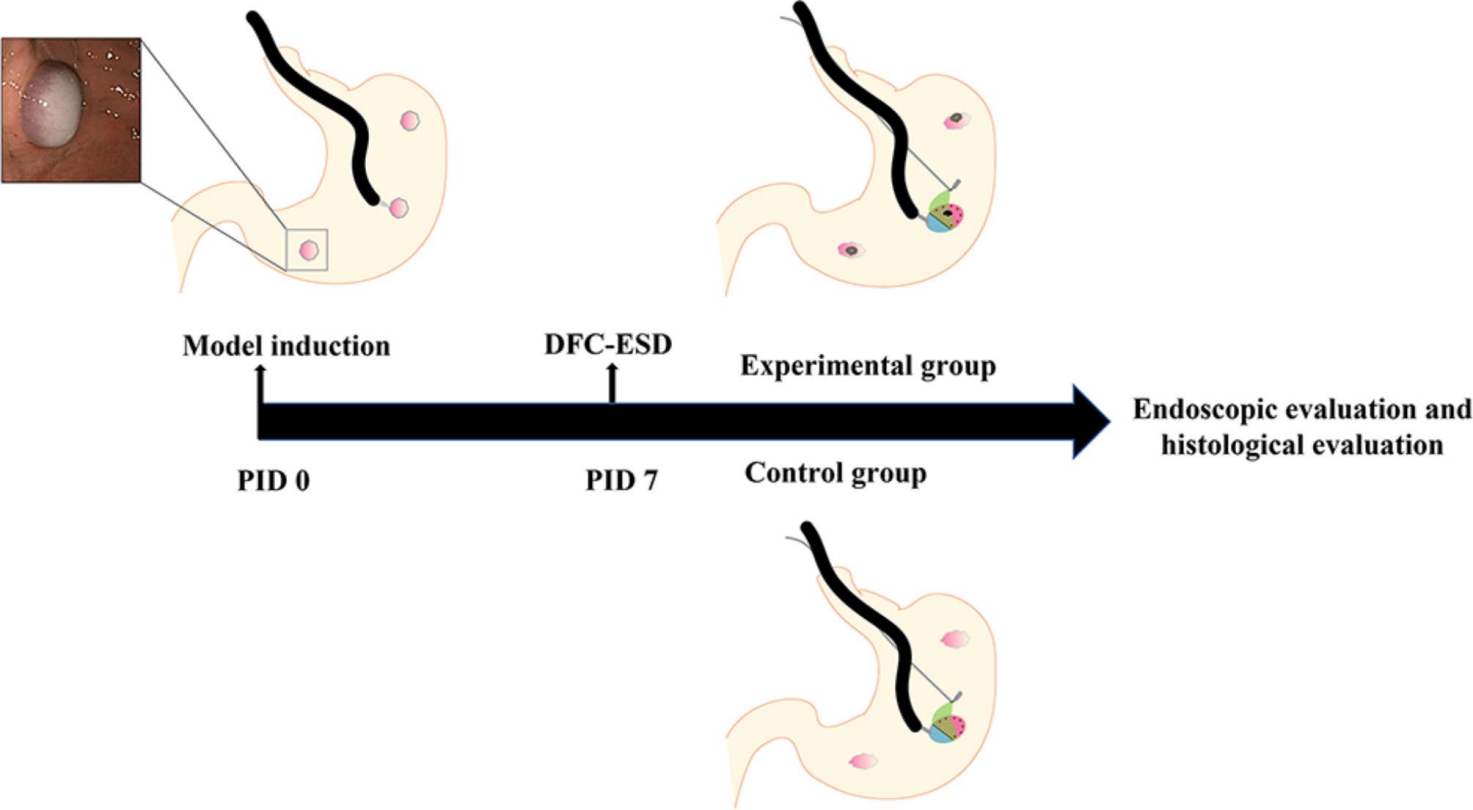
Schematic diagram of study protocol. Hypertonic dextrose solution (50%) was injected into submucosal layer at the fundus, body and antrum of gastric wall for induction of the fibrotic lesions. Dental floss clip traction assisted ESD (DFC-ESD) technique was performed in the experimental group and the control group respectively in PID 7. *PID*, Post-injection day; *DFC-ESD*, Dental floss clip traction assisted ESD

In order to create the simulated target lesions with local submucosal fibrosis in the experimental beagles, 2 to 3 mL of a 50% glucose solution were injected into the submucosal layer in the three predetermined places of the fundus, body, and antrum in the stomach. Within 30 min after injection of the 50% glucose solution, mucosal central whiteness with marginal redness at the injection area was evident [21]. On PID 7, endoscopic ultrasonography was performed to evaluate the presence of submucosal fibrosis formation or preoperative changes at the injection sites. Subsequently, the DFC-ESD was performed on the gastric local fibrotic lesion in the experimental beagles and on the normal gastric mucosa in the control beagles (Fig. 1).

### Dental floss clip traction assisted ESD (DFC-ESD) technique [8]

Two veteran endoscopists who had performed more than 1000 ESD procedures before this study carried out DFC-ESD in the gastric simulated target lesions. The major phases of DFC-ESD were similar to what previous study has described [22].

**Fig. 2:**
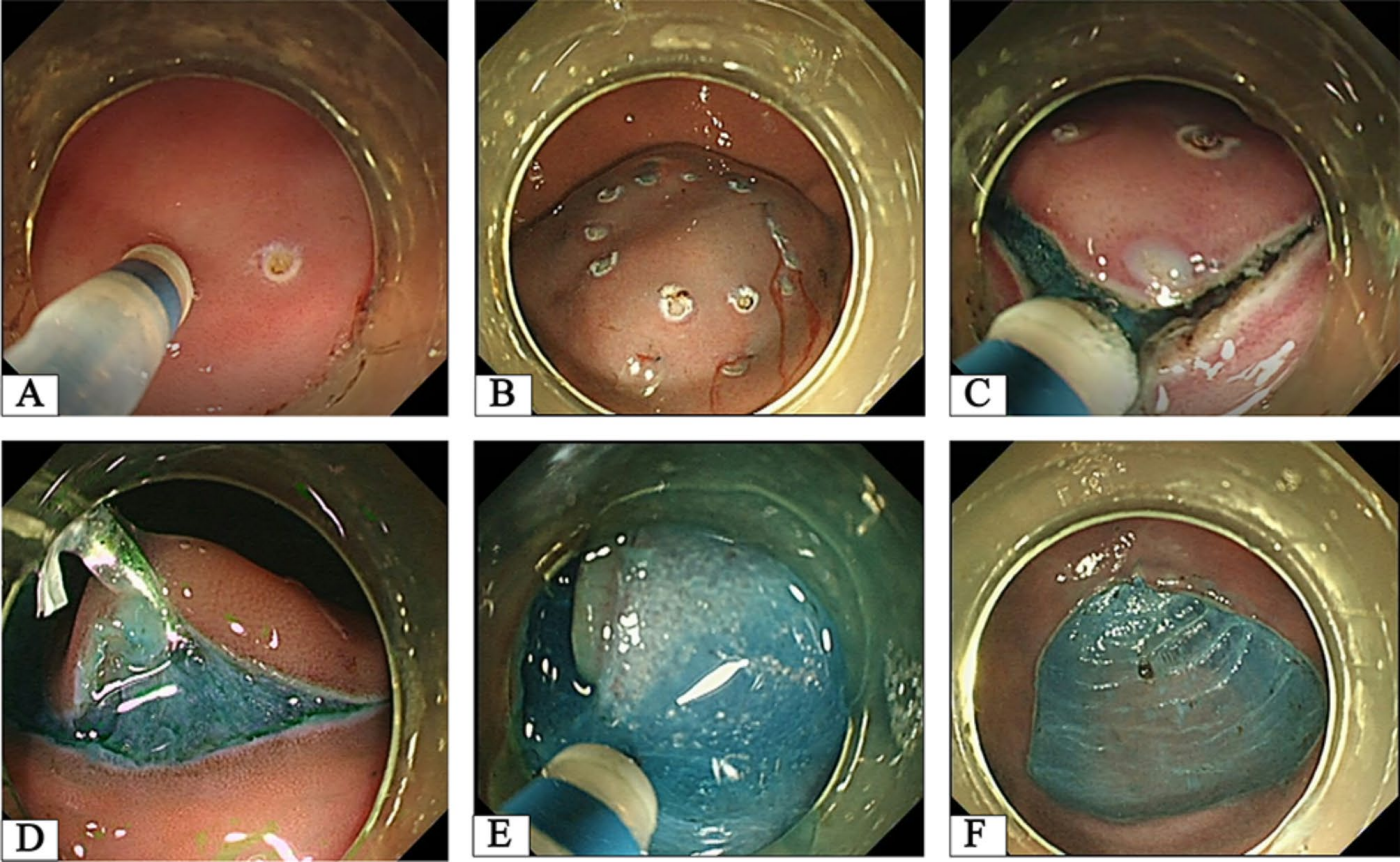
The major phases of DFC-ESD procedure. **A** Several dots were marked outside the margin of the target lesions. **B** The solution was injected into submucosal layer to create submucosal cushion. **C** The lesion was circumferential incision completely. **D** The endoscopic clip with the dental floss was deployed at the edge of mucosa for the traction system establishment. **E** Distinct submucosal layer exposure was achieved in virtue of dental floss clip traction. **F** The lesion was resected successfully without any iatrogenic adverse events

Following marking spots outside the target lesions and injecting submucosal solutions, circumferential incision was performed using a dual knife (KD-650 L; Olympus Optical Co., Ltd., Tokyo, Japan) (Fig. 2A-C). After the endoscope (GIF Q260J; Olympus Optical Co., Ltd., Tokyo, Japan) was removed, the clip device (ROCC-D-26-195; Nanjing Minimally Invasive Medical Technology Co., Ltd., China) was inserted into the channel of the endoscope. The endoscope was reinserted into the stomach after attaching the dental floss to the arm of the clip. Traction was successfully provided by dental floss after the clip was anchored to the edge of the mucosa (Fig. 2D). Thereafter, a precise cutting line was exposed so that careful dissection could be continued with the dual knife (Fig. 2E-F). And then the resected specimens were pinned on a foam board, photographed, and measured in size using ImageJ software (National Institutes of Health, Bethesda, MD, USA). After the procedures, two endoscopists filled out questionnaires on a 5-point scale from 0 (poor) to 5 (excellent) regarding their opinions on the realism and the feasibility of the animal model used for the evaluation and training of endoscopic techniques.

### Outcome measurement

The main variables we were analyzed: endoscopic observation, size of resected specimens, dissection time (minutes), dissection speed (cm^2^/min), amount of submucosal injection, en bloc resection rate, the frequency of severe hemorrhage and perforation, pathological evaluations, and the experts’ opinion scores.

For endoscopic assessment, submucosal fibrosis was evaluated by endoscopic ultrasonography, which presented a regionally thickened or bright submucosal layer compared to surrounding regions [23]. Dissection time was defined as the time interval between submucosal dissection and the completion of specimen resection. En bloc resection was the removal of the whole lesions in a single piece. Severe hemorrhage was defined as bleeding requiring control by electrical coagulation or closure of the bleeding site by the application of a hemostatic clip during the ESD procedure. Perforation was defined as the condition in which the gas and fluid in the stomach could pass directly into the abdominal cavity. For pathological evaluation, the resected tissues were fixed in 10% neutral buffered formalin, and all slices obtained from the center of the tissues were stained with hematoxylin & eosin, Masson trichrome and alpha-smooth muscle actin (αSMA). In addition, to evaluate the expression of αSMA-positive between two groups, we randomly photographed five fields (original magnification × 40) in the submucosa of the lesions for each section and quantitatively measured the average optical density for each field using the ImageJ software. The average optical density of five fields was averaged to estimate the expression of αSMA-positive in each section.

### Statistical analysis

All variables were reported as means with standard deviations or medians with ranges. The χ^2^ test or Fisher exact test was used to compare categorical variables, and the Student’s *t* test or the Mann-Whitney U-test was used to compare continuous variables. All analyses were performed using SPSS.26 software (IBM Corp.; Chicago, Illinois, USA) based on the data types. Statistical significance was set at a *P* value < 0.05.

## Results

### Animal general health

Twelve dogs involved were alive without any severe postoperative adverse events or deaths during the study. No changes in physiological status or food intake were observed in the animals after endoscopic performance. There was no statistical difference in the average weight between pre-operation day 1 and post-operation day 7 in the two groups.

### Endoscopic evaluation

**Fig. 3:**
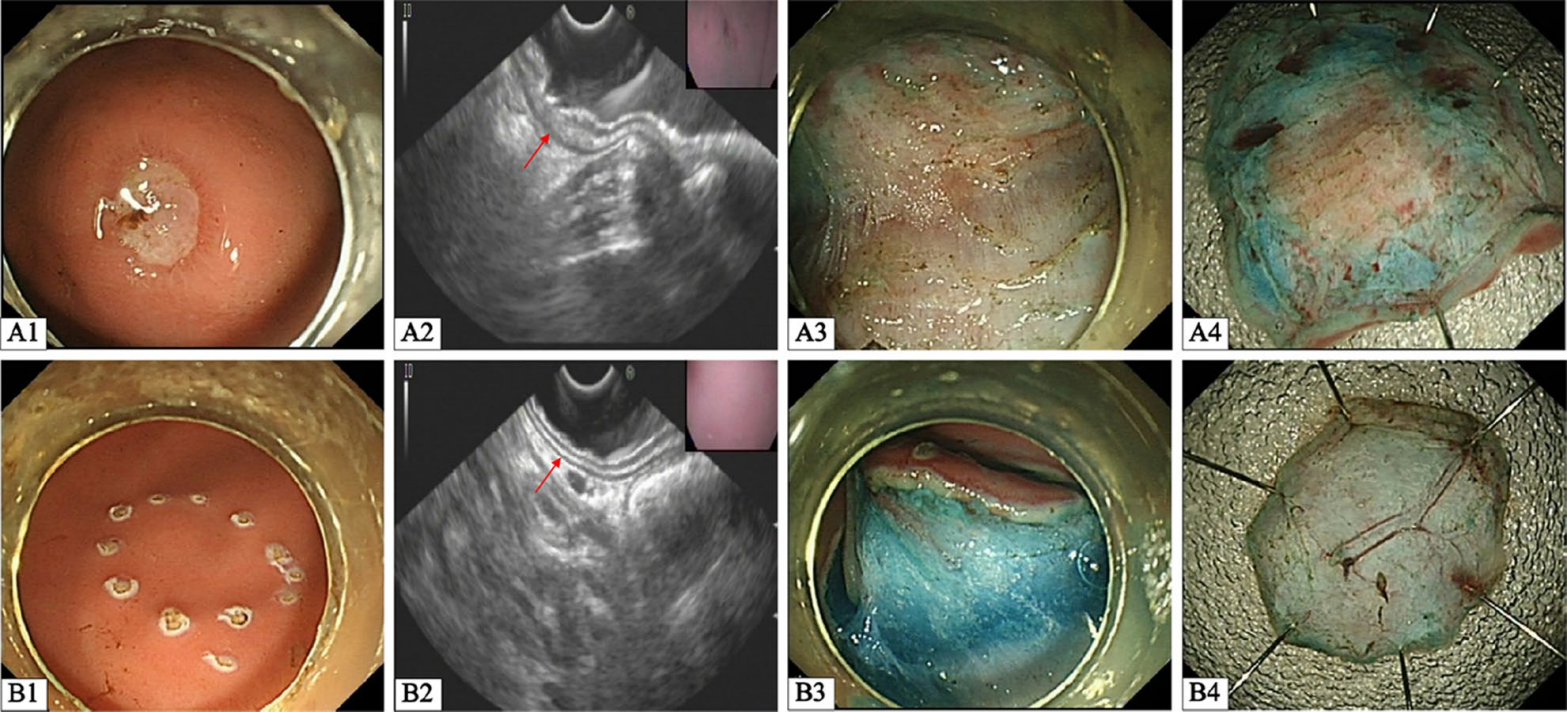
Endoscopic views of gastric lesions. **A** Gastric fibrotic target lesions. **B** Gastric normal target lesions. Compared with the normal gastric mucosa in the control group (**B1-2**), the center of injection area in the experimental group observed an ulceration covered with white mucoid cap on PID 7 (**A1**), with an endoscopic ultrasonography finding of locally interrupted and thickened submucosal layer (**A2, red arrow**). The submucosal layer showed moderate fibrosis in the experimental group, which appeared as a white web-like structure in the blue submucosal layer (**A3**), and the control group manifested as a blue transparent layer (**B3**). The section of resected specimen revealed scar-like tissue in the experimental group (**A4**), and the control group presented blue transparent submucosal layer with clear vessel (**B4**)

Endoscopic observation was illustrated in Fig. 3. One week after injection, the mucosa of 18 injection sites was depressed, an ulcer lesion covered with a white mucoid cap was formed in the 16 injection sites (16/18), and the surrounding mucosa was hyperemic and edema (Fig. 3A1). Endoscopic ultrasonography confirmed the local submucosal fibrosis formation in all ulcer lesions, with a sign of a local interrupted and thickened submucosal layer (Fig. 3A2). The mean size of the induced ulceration was 1.0 ± 0.2 cm^2^. Only mucosal erosion could be seen in the other 2 injection sites, but without ulcer lesion or local submucosal fibrosis formation. During dissection for the ulcer lesions, a white web-like structure appeared in the submucosal layer, closely connected with the muscular layer (Fig. 3A3). Turning the resected specimens over to the other side, scar-like tissues in the center of the section were revealed in the experimental group (Fig. 3A4). The gastric normal target lesions showed no structural tissue damage under endoscopic observation in the control group (Fig. 3B1-4).

### DFC-ESD procedure outcomes

In total, DFC-ESD procedures were successfully performed on the 32 gastric simulated target lesions by two endoscopists, and the outcomes were summarized in Table 1. The mean size of the resected specimens did not significantly differ between the two groups (3.9 ± 1.0 vs. 3.6 ± 0.2 cm^2^; *P* = 0.17). The mean submucosal dissection time was significantly longer in the experimental group than that in the control group (15.3 ± 5.6 vs. 6.8 ± 0.8 min; *P* < 0.001). The mean speed of dissection for the experimental group was slower than that for the control group (0.3 ± 0.1 vs. 0.5 ± 0.1 cm^2^/min; *P* < 0.001). Compared with the control group, the mean amount of submucosal injection fluid in the experimental group increased significantly (21.3 ± 2.5 vs. 15.6 ± 1.3 mL; *P* < 0.001). The rates of en bloc resection (13/16 vs. 16/16; *P* = 0.23), severe hemorrhage (2/16 vs. 0/16; *P* = 0.48), and perforation (1/16 vs. 0/16; *P* = 0.99) showed no significant difference between the gastric local fibrotic target lesions and the gastric normal target lesions. Only three lesions in the experimental group were removed in two pieces due to local submucosal fibrosis and intraoperative perforation.

**Table 1:** Comparison of ESD outcomes between experimental and control groups^†^

Outcomes	Experimental group (N = 16)	Control group (N = 16)	*P* value^‡^
Specimen size, cm^2^ (mean ± SD^§^)	3.9 ± 1.0	3.6 ± 0.2	0.17
Dissection time, min (mean ± SD)	15.3 ± 5.6	6.8 ± 0.8	< 0.001
Speed, cm^2^/min (mean ± SD)	0.3 ± 0.1	0.5 ± 0.1	< 0.001
Injection, mL (mean ± SD)	21.3 ± 2.5	15.6 ± 1.3	< 0.001
En bloc resection, yes/no	13/3	16/0	0.23
Severe hemorrhage, yes/no	2/14	0/16	0.48
Perforation, yes/no	1/15	0/16	0.99
Submucosal fibrosis, yes/no	16/0	0/16	< 0.001

^†^*P* value was calculated using the Fisher’s exact test for categorical data and Student’s t test for continuous data with normal distribution, respectively

^‡^*P* < 0.05

^§^SD, standard deviation

### Histologic pathology

**Fig. 4:**
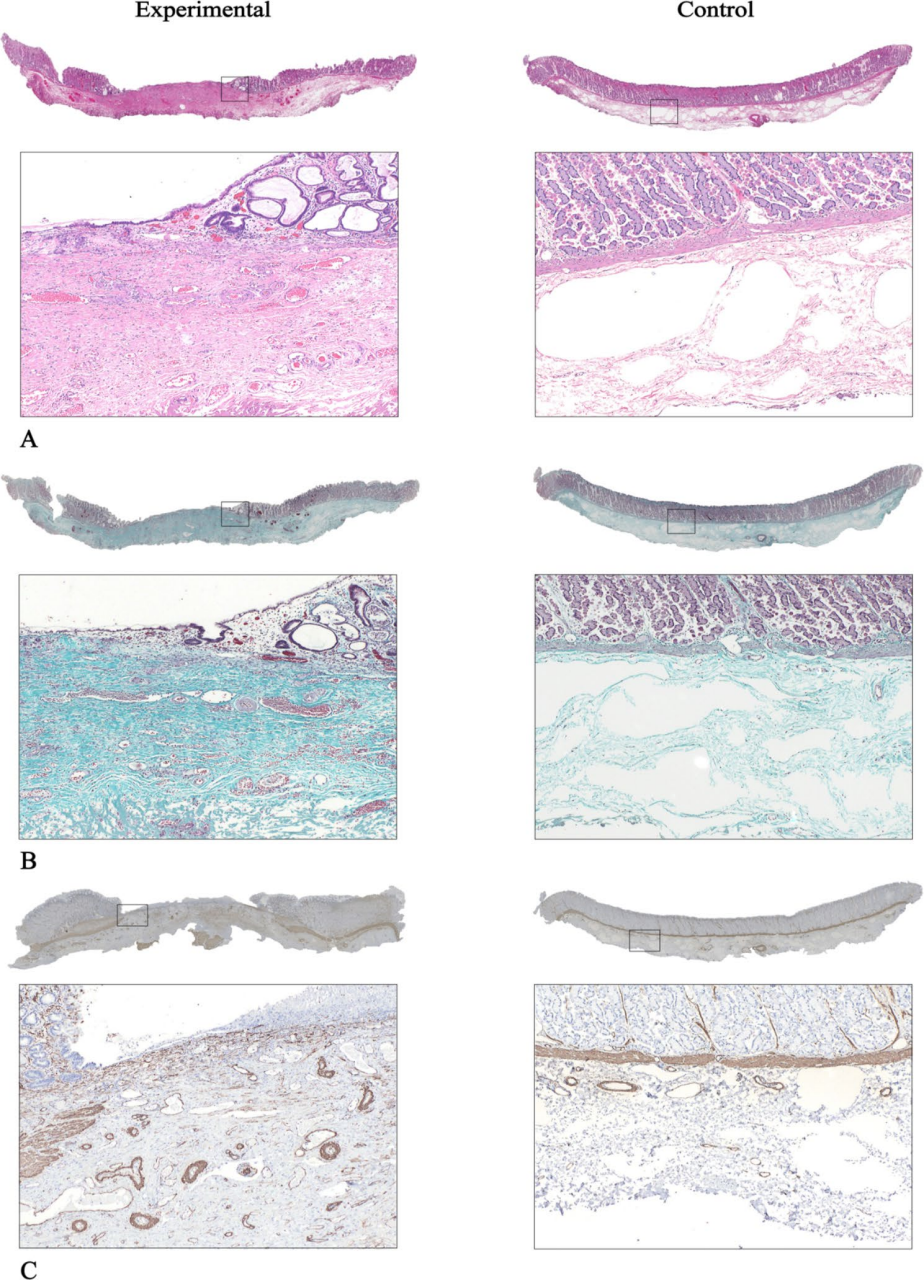
Histologic evaluation of hematoxylin & eosin, Masson trichrome staining and alpha-smooth muscle actin (αSMA). **A, B** Loss of epithelial tissue, inflammatory cell infiltration, vascular dilatation and congestion, and extensive submucosal fibrous tissue proliferations were observed in the experimental group (inset, original magnification, × 40). Masson trichrome staining of collagenous fiber (green). The control group represented regular arranged epithelial tissue and sparse connective tissue (inset, original magnification, × 40). **C** The αSMA-positive was intensively expressed in the submucosal layer of the experimental group than that in the control group on PID 7 (inset, original magnification, × 40). Immunohistochemical staining of αSMA protein (brown)

Hematoxylin & eosin and Masson trichrome staining of the gastric simulated lesions were presented in Fig. 4A-B. On PID 7 in the experimental group, the mucosa was completely eliminated in the center of the injection area, with inflammatory cell infiltration and vascular congestion, and ulceration with submucosal fibrosis was observed, occasionally with fibrinoid necrosis. Atypical regenerating epithelium with glandular proliferation and angiogenesis were observed in the surrounding mucosa (Fig. 4A). There was a striking difference in submucosal fibrosis proliferation between the experimental tissues and the normal gastric tissues stained by the Masson trichrome staining. Experimental tissues appeared to have plenty of intense green staining in the collagenous fiber deposition (Fig. 4B). The depth of dissection around the fibrotic portion was mostly limited to the muscle layer. The gastric normal gastric tissues showed no obvious histological changes in the control group (Fig. 4A-B).

The immunohistochemical findings of αSMA-positive between the gastric simulated target lesions were presented in Fig. 4C. The expression of αSMA-positive could be detected not only in fibroblasts and neovascularity but also in muscularis mucosae, blood vessel, and muscle layers. The αSMA-positive expression was more intense in the submucosal layer of the experimental group than that in the control group on PID 7, which indicated higher formation of fibroblasts and neovascularity (Fig. 4C). The expression of αSMA-positive in each section was quantitatively evaluated as the mean of average optical density. The mean of the average optical density was significantly higher in the experimental group than that in the control group (0.45 ± 0.02 vs. 0.49 ± 0.02, *P* < 0.001).

### Expert validation

The animal model had a great score for the realism of overall appearance of lesions (4.0 ± 0.0), structure around lesions (4.5 ± 0.7), margin identification (4.5 ± 0.7), submucosal fibrosis (4.5 ± 0.7), and adverse events (4.0 ± 0.0) when compared to those in the clinical conditions from the experts’ opinions. There were no differences in scores for the realism of submucosal injection, mucosal circumferential incision, or submucosal dissection (5.0 ± 0.0). The experts considered that the gastric simulated target lesions with local submucosal fibrosis were suitable for preclinical training and evaluation of endoscopic techniques (4.5 ± 0.7).

## Discussion

At present, ESD has become the preferred therapeutic approach to treat most endoscopically respectable gastrointestinal lesions [24], and it has been verified that it could be an effective therapy for residual or locally recurrent lesions after endoscopic resection [25, 26]. However, previous studies have proved that ulcerative scars and submucosal fibrosis are significant factors related to the technical difficulty of gastric ESD [27]. Even for a skilled endoscopist, the incidence of incomplete resection and adverse events in the fibrotic lesions is high [3, 28]. To date, several methods have been proposed to assist ESD procedures, some of which require preclinical evaluation and training. However, research on these new endoscopic techniques for lesions with submucosal fibrosis was seldom reported due to the lack of an optimal animal model.

Faller J et al. [25] also proposed that there was a specific learning curve for the local submucosal fibrotic lesions, and it was necessary for endoscopists to be trained in difficult ESD operation skills by a very professional endoscopic center. A proper animal model could be used for preclinical training and evaluating the effectiveness of new approaches to procedures [29]. Therefore, we proposed for the first time to establish a model of gastric local fibrotic target lesions in a live dog suitable for preclinical evaluation and training of new endoscopic techniques. Our study confirmed that the formation of submucosal fibrosis in the gastric lesions was similar to the clinical situation and could increase the technological difficulty and prolong the operative time. In the animal model we developed, we found that the DFC-ESD technique could also successfully remove lesions with local submucosal fibrosis, which may be an effective and safe method for such gastric lesions.

We induced ulcer lesions by injecting a 50% glucose solution into the submucosa of the stomach, with local submucosal fibrosis formation on endoscopic ultrasonography. And the blue submucosal layer appeared to have a white web-like structure during dissection, which could be divided into moderate fibrosis based on the submucosal fibrosis classification system proposed by Matsumoto A [30]. Histological analysis of the ulcerative lesions revealed the absence of epithelial cells, inflammatory cell infiltration, and the deposition of submucosal collagen. This kind of pathophysiological change may be caused by the osmotic pressure difference between the inside and outside of the cell membrane after injecting hypertonic sugar into the submucosa, resulting in tissue damage that activates myofibroblasts and secretes collagen fibers, leading to the proliferation of mature fibrous tissues. Based on the endoscopic and histological evaluation, one week after injection may be the appropriate time to start the technique training project.

In the previous study, target lesions like pseudopolyps or depressed mucosal defects without submucosal fibrosis were considered as training models [20, 31]. Esaki M et al. [32] proposed an artificial esophagus model with fibrosis created at the center of the mock lesion by thread suturing and proved that it could be used to evaluate the usefulness of clip-with-thread traction for difficult ESD. However, the artificial fibrosis model was short on blood supply compared with the simulated lesions we developed, which could not evaluate the influence of bleeding during ESD performance. The simulated target lesions in our study were easy to reproduce, did no harm to the animal health, and had high feasibility as a learning tool.

We found that the dissection time of DFC-ESD was about 2 times longer in the gastric fibrotic lesions compared to the normal one. In fact, in order to perform lesions with submucosal fibrosis successfully, appropriate traction needed to be done to separate the submucosal layer from the muscle layer until a precise cutting line was exposed. Even if the traction system was established, dissection toward the fibrotic portion with the dual knife still needed to be cautious and slow. In addition, the submucosal fibrosis also increased the amount of submucosal injection fluid needed to elevate the lesion, because normal saline solution may not maintain adequate submucosal elevation in the lesions with submucosal fibrosis. For fibrotic lesions, the high viscosity and mechanical dissection properties of submucosal injections may be a better choice to improve the submucosal lifting effect [25, 33, 34]. These facts showed that the presented model could simulate a more complex condition to evaluate and train new therapeutic endoscopic methods.

It was reported that fibrotic lesions had a higher frequency of complications in clinical practice due to the interaction of inflammatory and operative factors, notably bleeding and incomplete resection [3, 35]. This is primarily because of submucosal fibrosis formation with no accurate anatomical line, leading to incremental difficulty in operation. While there was no significant difference in the rate of adverse events between the two groups in our study, mainly because the dental floss clip traction system enhanced direct visualization of the submucosal layer in the fibrotic lesions and reduced the risks of adverse events. Besides, we could manage the hemorrhage and perforation by endoscopic treatment, which did no harm to animal health and could not lead to any irretrievable results. Thus, we considered that the dental floss clip traction system may be an effective way to assist ESD with such difficult lesions. However, the effectiveness and safety of the DFC-ESD technique in gastric fibrotic lesions still need to be further analyzed in the following studies. And more endoscopic techniques could be evaluated and trained by this model to promote the advancement of endoscopy.

This study has some limitations which should be acknowledged. Although the beagle model represented gastric difficult lesions similar to clinical situations with its inflammatory reaction and submucosal fibrosis formation, it did not have the properties of early tumor. In addition, mock lesions by injecting 50% dextrose water (DW) could not reproduce all categories of submucosal fibrosis. In the preliminary experiment, we also attempted different concentrations of DW; however, the lower the concentration of DW injecting into the submucosal layer, the lower the incidence of submucosal fibrosis. We are planning to conduct more experiments to determine optimal concentrations of solution in different locations of gastrointestinal tract.

In conclusion, we developed a novel animal model of gastric target lesions with local submucosal fibrosis, and it strongly appeared to be suitable for preclinical evaluation and training of new endoscopic techniques. In the future, large-scale experiments with an appropriate sample of trainees and assessments of experimental data will need to be conducted to verify its clinical value.

### Electronic supplementary material

Below is the link to the electronic supplementary material.

**Supplementary Material 1:** CONSORT 2010 checklist of information to include when reporting a randomised trial

## Data Availability

The datasets used and analyzed during the current study are available from the corresponding author on reasonable request.

## Abbreviations

αSMA: Alpha smooth muscle actin
DFC-ESD: Dental floss clip traction assisted ESD technique
DW: Dextrose water
ESD: Endoscopic submucosal dissection
PID: Post-injection day
